# Supplementary material for: Non-Hermitian non-equipartition theory for trapped particles
Source: Nat Commun. 2024 Mar 4;15:1963. doi: 10.1038/s41467-024-46058-5 (PMC10912716; doi:10.1038/s41467-024-46058-5)
Supplement: Supplementary file 1 — Supplementary Information [file 41467_2024_46058_MOESM1_ESM.pdf]

Supplementary Information

for

“Non-Hermitian Non-Equipartition Theory for Trapped Particles”

Xiao Li[1,2]†, Yongyin Cao[3]†, and Jack Ng[1]*

[1]*Department of Physics, Southern University of Science and Technology,*
Shenzhen, Guangdong 518055, China.

[2]*Department of Physics, The Hong Kong University of Science and Technology,*
Hong Kong, China.

[3]*Institute of Advanced Photonics, School of Physics, Harbin Institute of Technology,*
Harbin 150001, China.

†These authors contributed equally.

*E-mail: wuzh3@sustech.edu.cn

Supplementary Note 1: Derivation of the non-Hermitian non-equipartition (NHNE) theory in two-dimensional (2D) space

We derive the explicit expression that generalizes the well-known equipartition theorem (ET) to a form that can describe particles trapped by a non-Hermitian force. We call this the non-Hermitian non-equipartition (NHNE) theory. Our results show that the ET fails utterly to describe such particles, and the average energy for each quadratic degree of freedom is neither $k_B T/2$ nor equipartitioned.

Consider a particle immersed in a fluid of temperature T and subject to an external non-Hermitian force $\mathbf{F}(\mathbf{r}=(x,y,z))=(F_x, F_y, F_z)$. Such an external force can be optical, acoustic, or any other similar force. The dynamics of the particle are governed by the Langevin stochastic differential equation [i]:

$$m\frac{d\mathbf{v}}{dt}=\mathbf{F}(\mathbf{r})-\gamma\mathbf{v}+\mathbf{A}(t), \tag{S.1}$$

where m is the particle mass, $\mathbf{v}=(v_x, v_y, v_z)$ is the particle velocity, $\gamma=6\pi\eta a$ is the friction coefficient of a sphere with radius a and $\mathbf{A}(t)=(A_x(t), A_y(t), A_z(t))$ is the Gaussian-distributed random fluctuating force with correlations of

$$\langle A_i(t)A_j(t')\rangle = 2\gamma k_B T\delta_{i,j}\delta(t-t'), \tag{S.2}$$

where $A_i(t)$ is the ith Cartesian component of $\mathbf{A}(t)$, $\delta_{i,j}$ is the Kronecker delta function, and $\delta(t)$ is the Dirac delta function. The coefficient on the right-hand side of Eq. (S.2) is determined by the fluctuation–dissipation theorem.

At a mechanical equilibrium located at $\mathbf{r}=\mathbf{0}$, $\mathbf{F}(\mathbf{0})$ vanishes. Thus, in the vicinity of the trapping center, the force may be approximated by the linear term of its Taylor series, namely

$$\mathbf{F}(\mathbf{r})\approx\overleftrightarrow{\mathbf{K}}\cdot\mathbf{r}, \tag{S.3}$$

where

$$\overleftrightarrow{\mathbf{K}} = \begin{bmatrix} \frac{\partial F_x}{\partial x} & \frac{\partial F_x}{\partial y} & \frac{\partial F_x}{\partial z} \\ \frac{\partial F_y}{\partial x} & \frac{\partial F_y}{\partial y} & \frac{\partial F_y}{\partial z} \\ \frac{\partial F_z}{\partial x} & \frac{\partial F_z}{\partial y} & \frac{\partial F_z}{\partial z} \end{bmatrix} = \begin{bmatrix} k_{xx} & k_{xy} & k_{xz} \\ k_{yx} & k_{yy} & k_{yz} \\ k_{zx} & k_{zy} & k_{zz} \end{bmatrix}, \tag{S.4}$$

is the force matrix. The Langevin equation is reduced to

$$m\frac{d\mathbf{v}}{dt} = \overleftrightarrow{\mathbf{K}} \cdot \mathbf{r} - \gamma \mathbf{v} + \mathbf{A}(t). \tag{S.5}$$

Equation (S.5) determines the dynamics and the stability of the particle near the mechanical equilibrium.

In the special case in which the external force is conservative, the real matrix $\overleftrightarrow{\mathbf{K}}$ is Hermitian and symmetric ($\overleftrightarrow{\mathbf{K}} = \overleftrightarrow{\mathbf{K}}^{\dagger} = \overleftrightarrow{\mathbf{K}}^{\mathrm{T}}$). The ET holds, and each quadratic degree of freedom has an average energy of $k_B T/2$ [i]. However, if the external force is non-conservative, as is the case for an optical or acoustic force, $\overleftrightarrow{\mathbf{K}}$ is a non-Hermitian matrix ($\overleftrightarrow{\mathbf{K}} \neq \overleftrightarrow{\mathbf{K}}^{\dagger}$), and the ET fails utterly.

To show this, we first analytically derive the NHNE theory for a simpler 2D problem, where the z-motion is decoupled from the transverse motion:

$$\overleftrightarrow{\mathbf{K}} = \begin{bmatrix} k_{xx} & k_{xy} & 0 \\ k_{yx} & k_{yy} & 0 \\ 0 & 0 & k_{zz} \end{bmatrix}. \tag{S.6}$$

As discussed in the main text, Eq. (S.6) is valid for several types of common beams used in optical trapping. The force matrix in Eq. (S.6) is non-Hermitian when $k_{xy} \neq k_{yx}$, which occurs when the beam possesses angular momentum [ii]. It is legitimate to focus on the following 2 × 2 matrix for the transverse motion:

$$\overleftrightarrow{\mathbf{K}}_{\mathrm{2D}} = \begin{bmatrix} k_{xx} & k_{xy} \\ k_{yx} & k_{yy} \end{bmatrix}. \tag{S.7}$$

Equation (S.5) then becomes

$$m\frac{d\mathbf{v}}{dt} = \overleftrightarrow{\mathbf{K}}_{\mathrm{2D}} \cdot \mathbf{r} - \gamma\mathbf{v} + \mathbf{A}(t)\,, \tag{S.8}$$

where $\mathbf{r} = \begin{bmatrix} r_1 \\ r_2 \end{bmatrix} = \begin{bmatrix} x \\ y \end{bmatrix}$, $\mathbf{v} = \begin{bmatrix} v_1 \\ v_2 \end{bmatrix} = \begin{bmatrix} v_x \\ v_y \end{bmatrix}$, and $\mathbf{A}(t) = \begin{bmatrix} A_1(t) \\ A_2(t) \end{bmatrix} = \begin{bmatrix} A_x(t) \\ A_y(t) \end{bmatrix}$.

By diagonalizing $-\overleftrightarrow{\mathbf{K}}_{\mathrm{2D}}/m$ using $\overleftrightarrow{\mathbf{\Lambda}}$, which is formed by using its right eigenvectors as columns, Eq. (S.8) becomes

$$d\mathbf{u}/dt = -\overleftrightarrow{\mathbf{K}}' \cdot \mathbf{q} - \beta\mathbf{u} + \mathbf{A}'(\mathbf{q},t)\,, \tag{S.9}$$

where $\mathbf{q} = \overleftrightarrow{\mathbf{\Lambda}}^{-1} \cdot \mathbf{r}$, $\mathbf{u} = \overleftrightarrow{\mathbf{\Lambda}}^{-1} \cdot \mathbf{v}$, $\mathbf{A}'(\mathbf{q},t) = \overleftrightarrow{\mathbf{\Lambda}}^{-1} \cdot \mathbf{A}(\mathbf{r},t)/m$, $\beta = \gamma/m$, and $\overleftrightarrow{\mathbf{K}}' = \overleftrightarrow{\mathbf{\Lambda}}^{-1}\left(-\overleftrightarrow{\mathbf{K}}_{\mathrm{2D}}/m\right)\overleftrightarrow{\mathbf{\Lambda}}$ is a diagonalized matrix. The ith eigenfrequency is given by $\omega_i = \sqrt{K_i}$, where K_i is the ith eigenvalue of $-\overleftrightarrow{\mathbf{K}}_{\mathrm{2D}}/m$. The eigenfrequencies are generally complex numbers, as K_i can be a complex number when $-\overleftrightarrow{\mathbf{K}}_{\mathrm{2D}}/m$ is non-Hermitian. Equation (S.9) is now decoupled:

$$\begin{bmatrix} du_1/dt \\ du_2/dt \end{bmatrix} = -\begin{bmatrix} \omega_1^2 & 0 \\ 0 & \omega_2^2 \end{bmatrix}\begin{bmatrix} q_1 \\ q_2 \end{bmatrix} - \beta\begin{bmatrix} u_1 \\ u_2 \end{bmatrix} + \begin{bmatrix} A_1'(t) \\ A_2'(t) \end{bmatrix}, \tag{S.10}$$

where $\mathbf{q} = \begin{bmatrix} q_1 \\ q_2 \end{bmatrix}$ and $\mathbf{u} = \begin{bmatrix} u_1 \\ u_2 \end{bmatrix}$ are the generalized coordinate and generalized velocity, respectively. Following ref. [i], the solution of Eq. (S.10) is

$$\begin{cases} q_1 + \dfrac{1}{\mu_1^+ - \mu_1^-}\left[(q_{01}\mu_1^- - u_{01})e^{\mu_1^+ t} - (q_{01}\mu_1^+ - u_{01})e^{\mu_1^- t}\right] = \int_0^t A_1'(\tau)\varphi_1(\tau)d\tau, \\ q_2 + \dfrac{1}{\mu_2^+ - \mu_2^-}\left[(q_{02}\mu_2^- - u_{02})e^{\mu_2^+ t} - (q_{02}\mu_2^+ - u_{02})e^{\mu_2^- t}\right] = \int_0^t A_2'(\tau)\varphi_2(\tau)d\tau, \end{cases} \tag{S.11}$$

$$\begin{cases} u_1 + \dfrac{1}{\mu_1^+ - \mu_1^-}\left[\mu_1^+(q_{01}\mu_1^- - u_{01})e^{\mu_1^+ t} - \mu_1^-(q_{01}\mu_1^+ - u_{01})e^{\mu_1^- t}\right] = \int_0^t A_1'(\tau)\phi_1(\tau)d\tau, \\ u_2 + \dfrac{1}{\mu_2^+ - \mu_2^-}\left[\mu_2^+(q_{02}\mu_2^- - u_{02})e^{\mu_2^+ t} - \mu_2^-(q_{02}\mu_2^+ - u_{02})e^{\mu_2^- t}\right] = \int_0^t A_2'(\tau)\phi_2(\tau)d\tau, \end{cases} \tag{S.12}$$

where

$$\varphi_i(\tau) = \frac{1}{\mu_i^+ - \mu_i^-}\left(e^{\mu_i^+(t-\tau)} - e^{\mu_i^-(t-\tau)}\right), \tag{S.13}$$

$$\phi_i(\tau) = \frac{1}{\mu_i^+ - \mu_i^-}\left(\mu_i^+ e^{\mu_i^+(t-\tau)} - \mu_i^- e^{\mu_i^-(t-\tau)}\right), \tag{S.14}$$

and

$$\mu_i^\pm = \frac{1}{2}\left(-\beta \pm \sqrt{\beta^2 - 4\omega_i^2}\right), \tag{S.15}$$

with i = 1 or 2. Here, $\mathbf{q}_0 = \begin{bmatrix} q_{01} \\ q_{02} \end{bmatrix}$ and $\mathbf{u}_0 = \begin{bmatrix} u_{01} \\ u_{02} \end{bmatrix}$ are the initial conditions for the generalized displacement and velocity, respectively.

After rearranging Eq. (S.11), we obtain

$$\begin{bmatrix} q_1 \\ q_2 \end{bmatrix} + \begin{bmatrix} b_1^+ + b_1^- & 0 \\ 0 & b_1^+ - b_1^- \end{bmatrix}\begin{bmatrix} q_{01} \\ q_{02} \end{bmatrix} - \begin{bmatrix} b_2^+ + b_2^- & 0 \\ 0 & b_2^+ - b_2^- \end{bmatrix}\begin{bmatrix} u_{01} \\ u_{02} \end{bmatrix} = \int_0^t \begin{bmatrix} A_1'(\tau)\varphi_1(\tau) \\ A_2'(\tau)\varphi_2(\tau) \end{bmatrix} d\tau, \tag{S.16}$$

where

$$\begin{cases} b_1^\pm = \dfrac{1}{2}\left(\dfrac{\mu_1^- e^{\mu_1^+ t} - \mu_1^+ e^{\mu_1^- t}}{\mu_1^+ - \mu_1^-} \pm \dfrac{\mu_2^- e^{\mu_2^+ t} - \mu_2^+ e^{\mu_2^- t}}{\mu_2^+ - \mu_2^-}\right), \\ b_2^\pm = \dfrac{1}{2}\left(\dfrac{e^{\mu_1^+ t} - e^{\mu_1^- t}}{\mu_1^+ - \mu_1^-} \pm \dfrac{e^{\mu_2^+ t} - e^{\mu_2^- t}}{\mu_2^+ - \mu_2^-}\right). \end{cases} \tag{S.17}$$

Now, we can transform Eq. (S.16) back to the Cartesian coordinate system by multiplying by $\boldsymbol{\Lambda}^{-1}$ from the left:

$$\begin{aligned} &\overleftrightarrow{\boldsymbol{\Lambda}} \cdot \begin{bmatrix} q_1 \\ q_2 \end{bmatrix} + \overleftrightarrow{\boldsymbol{\Lambda}}\begin{bmatrix} b_1^+ + b_1^- & 0 \\ 0 & b_1^+ - b_1^- \end{bmatrix}\overleftrightarrow{\boldsymbol{\Lambda}}^{-1}\overleftrightarrow{\boldsymbol{\Lambda}} \cdot \begin{bmatrix} q_{01} \\ q_{02} \end{bmatrix} - \overleftrightarrow{\boldsymbol{\Lambda}}\begin{bmatrix} b_2^+ + b_2^- & 0 \\ 0 & b_2^+ - b_2^- \end{bmatrix}\overleftrightarrow{\boldsymbol{\Lambda}}^{-1}\overleftrightarrow{\boldsymbol{\Lambda}} \cdot \begin{bmatrix} u_{01} \\ u_{02} \end{bmatrix} \\ &\qquad = \int_0^t \overleftrightarrow{\boldsymbol{\Lambda}}\begin{bmatrix} \varphi_1(\tau) & 0 \\ 0 & \varphi_2(\tau) \end{bmatrix}\overleftrightarrow{\boldsymbol{\Lambda}}^{-1}\overleftrightarrow{\boldsymbol{\Lambda}} \cdot \begin{bmatrix} A_1'(\tau) \\ A_2'(\tau) \end{bmatrix} d\tau. \end{aligned} \tag{S.18}$$

Thus, after rearrangement, we obtain

$$
\mathbf{r}+\begin{bmatrix} b_1^+ - \alpha(k_{xx}-k_{yy})b_1^- & -2\alpha k_{xy}b_1^- \\ -2\alpha k_{yx}b_1^- & b_1^+ + \alpha(k_{xx}-k_{yy})b_1^- \end{bmatrix}\mathbf{r}_0 - \begin{bmatrix} b_2^+ - \alpha(k_{xx}-k_{yy})b_2^- & -2\alpha k_{xy}b_2^- \\ -2\alpha k_{yx}b_2^- & b_2^+ + \alpha(k_{xx}-k_{yy})b_2^- \end{bmatrix}\mathbf{v}_0
$$
$$
=\int_0^t \overleftrightarrow{\mathbf{\Lambda}}\begin{bmatrix} \varphi_1(\tau) & 0 \\ 0 & \varphi_2(\tau) \end{bmatrix}\overleftrightarrow{\mathbf{\Lambda}}^{-1}\frac{1}{m}\begin{bmatrix} A_1(\tau) \\ A_2(\tau) \end{bmatrix}d\tau
$$
$$
=\int_0^t \frac{1}{m}\begin{bmatrix} \varphi^+(\tau)-\alpha(k_{xx}-k_{yy})\varphi^-(\tau) & -2\alpha k_{xy}\varphi^-(\tau) \\ -2\alpha k_{yx}\varphi^-(\tau) & \varphi^+(\tau)+\alpha(k_{xx}-k_{yy})\varphi^-(\tau) \end{bmatrix}\begin{bmatrix} A_1(\tau) \\ A_2(\tau) \end{bmatrix}d\tau,
$$
(S.19)

where

$$
\alpha = 1\Big/\sqrt{(k_{xx}-k_{yy})^2+4k_{xy}k_{yx}}\ , \tag{S.20}
$$

and

$$
\varphi^{\pm}(\tau)=\frac{1}{2}\left(\varphi_1(\tau)\pm\varphi_2(\tau)\right). \tag{S.21}
$$

where $\mathbf{r}_0=\begin{bmatrix} x_0 \\ y_0 \end{bmatrix}$ and $\mathbf{v}_0=\begin{bmatrix} v_{0x} \\ v_{0y} \end{bmatrix}$ are the initial displacement and velocity in Cartesian coordinates, respectively.

To simplify notation, we denote the left-hand side of Eq. (S.19) as

$$
\begin{cases} R_1 = x+[b_1^+-\alpha(k_{xx}-k_{yy})b_1^-]x_0-2\alpha k_{xy}b_1^-y_0-[b_2^+-\alpha(k_{xx}-k_{yy})b_2^-]v_{0x}+2\alpha k_{xy}b_2^-v_{0y}, \\ R_2 = y-2\alpha k_{yx}b_1^-x_0+[b_1^++\alpha(k_{xx}-k_{yy})b_1^-]y_0+2\alpha k_{yx}b_2^-v_{0x}-[b_2^++\alpha(k_{xx}-k_{yy})b_2^-]v_{0y}, \end{cases} \tag{S.22}
$$

and then we have

$$
\begin{cases} R_1 = \int_0^t\left[\left(\varphi^+(\tau)-\alpha(k_{xx}-k_{yy})\varphi^-(\tau)\right)\frac{A_1(\tau)}{m}-2\alpha k_{xy}\varphi^-(\tau)\frac{A_2(\tau)}{m}\right]d\tau, \\ R_2 = \int_0^t\left[\left(\varphi^+(\tau)+\alpha(k_{xx}-k_{yy})\varphi^-(\tau)\right)\frac{A_2(\tau)}{m}-2\alpha k_{yx}\varphi^-(\tau)\frac{A_1(\tau)}{m}\right]d\tau. \end{cases} \tag{S.23}
$$

By dividing the integration interval [0, t] into a large number of subintervals, each with a duration of Δt, Eq. (S.23) becomes

$$\begin{cases} R_1 = \sum_j \left(\varphi^+(j\Delta t) - \alpha(k_{xx}-k_{yy})\varphi^-(j\Delta t)\right)\int_{j\Delta t}^{(j+1)\Delta t} \frac{A_1(\tau)}{m} d\tau - 2\alpha k_{xy}\varphi^-(j\Delta t)\int_{j\Delta t}^{(j+1)\Delta t} \frac{A_2(\tau)}{m} d\tau, \\ R_2 = \sum_j \left(\varphi^+(j\Delta t) + \alpha(k_{xx}-k_{yy})\varphi^-(j\Delta t)\right)\int_{j\Delta t}^{(j+1)\Delta t} \frac{A_2(\tau)}{m} d\tau - 2\alpha k_{yx}\varphi^-(j\Delta t)\int_{j\Delta t}^{(j+1)\Delta t} \frac{A_1(\tau)}{m} d\tau. \end{cases}$$

(S.24)

Here, we define the impulse transferred to the Brownian particle during Δt as

$$B_i(\Delta t) = \int_{j\Delta t}^{(j+1)\Delta t} A_i(\tau) d\tau \,, \tag{S.25}$$

The distribution of $B_i(\Delta t)$ is governed by the distribution function, that is

$$W\left[B_i(\Delta t)\right] = \frac{1}{\sqrt{2\pi\sigma_i^2}} e^{-\frac{|B_i(\Delta t)|^2}{2\sigma_i^2}} \,, \tag{S.26}$$

where $\sigma_i^2 = 2\beta m k_{\mathrm{B}} T \Delta t$ is the variance of $B_i(\Delta t)$. As the mean of $B_i(\Delta t)$ is 0, $\langle R_1 \rangle = \langle R_2 \rangle = 0$. Thus, from Eq. (S.22), we obtain

$$\begin{cases} \langle x \rangle = -[b_1^+ - \alpha(k_{xx}-k_{yy})b_1^-]x_0 + 2\alpha k_{xy} b_1^- y_0 + [b_2^+ - \alpha(k_{xx}-k_{yy})b_2^-]v_{0x} - 2\alpha k_{xy} b_2^- v_{0y}, \\ \langle y \rangle = 2\alpha k_{yx} b_1^- x_0 - [b_1^+ + \alpha(k_{xx}-k_{yy})b_1^-]y_0 - 2\alpha k_{yx} b_2^- v_{0x} + [b_2^+ + \alpha(k_{xx}-k_{yy})b_2^-]v_{0y}. \end{cases} \tag{S.27}$$

If we write Eq. (S.24) as

$$\begin{cases} R_1 = \sum_j r_1^j, \\ R_2 = \sum_j r_2^j, \end{cases} \tag{S.28}$$

with

$$\begin{cases} r_1^j = \left(\varphi^+(j\Delta t) - \alpha(k_{xx}-k_{yy})\varphi^-(j\Delta t)\right)\int_{j\Delta t}^{(j+1)\Delta t} \frac{A_1(\tau)}{m} d\tau - 2\alpha k_{xy}\varphi^-(j\Delta t)\int_{j\Delta t}^{(j+1)\Delta t} \frac{A_2(\tau)}{m} d\tau, \\ r_2^j = \left(\varphi^+(j\Delta t) + \alpha(k_{xx}-k_{yy})\varphi^-(j\Delta t)\right)\int_{j\Delta t}^{(j+1)\Delta t} \frac{A_2(\tau)}{m} d\tau - 2\alpha k_{yx}\varphi^-(j\Delta t)\int_{j\Delta t}^{(j+1)\Delta t} \frac{A_1(\tau)}{m} d\tau, \end{cases}$$

(S.29)

the variances of r_1^j and r_2^j are

$$\begin{cases} \sigma_{r_1^j}^2 = \dfrac{2\beta k_{\mathrm{B}} T \Delta t}{m}\left[\left(\varphi^+(j\Delta t) - \alpha(k_{xx}-k_{yy})\varphi^-(j\Delta t)\right)^2 + \left(2\alpha k_{xy}\varphi^-(j\Delta t)\right)^2\right], \\ \sigma_{r_2^j}^2 = \dfrac{2\beta k_{\mathrm{B}} T \Delta t}{m}\left[\left(\varphi^+(j\Delta t) + \alpha(k_{xx}-k_{yy})\varphi^-(j\Delta t)\right)^2 + \left(2\alpha k_{yx}\varphi^-(j\Delta t)\right)^2\right], \end{cases} \tag{S.30}$$

as $B_1(\Delta t)$ and $B_2(\Delta t)$ are uncorrelated. We can also write the probability distribution of r_i^j:

$$W\left[r_i^j\right] = \frac{1}{\sqrt{2\pi\sigma_{r_i^j}^2}} e^{-\frac{\left|r_i^j\right|^2}{2\sigma_{r_i^j}^2}}, \tag{S.31}$$

and the probability distribution of R_i:

$$W\left[R_i\right] = \frac{1}{\sqrt{2\pi\sum_j \sigma_{r_i^j}^2}} e^{-\frac{\left|R_i\right|^2}{2\sum_j \sigma_{r_i^j}^2}}, \tag{S.32}$$

where

$$\begin{cases} \sum_j \sigma_{r_1^j}^2 = \dfrac{2\beta k_{\mathrm{B}} T}{m}\int_0^t\left[\left(\varphi^+(\tau) - \alpha(k_{xx}-k_{yy})\varphi^-(\tau)\right)^2 + \left(2\alpha k_{xy}\varphi^-(\tau)\right)^2\right]d\tau, \\ \sum_j \sigma_{r_2^j}^2 = \dfrac{2\beta k_{\mathrm{B}} T}{m}\int_0^t\left[\left(\varphi^+(\tau) + \alpha(k_{xx}-k_{yy})\varphi^-(\tau)\right)^2 + \left(2\alpha k_{yx}\varphi^-(\tau)\right)^2\right]d\tau, \end{cases} \tag{S.33}$$

which in turn gives

$$\begin{cases} \left\langle x^2\right\rangle = \left\langle x\right\rangle^2 + \dfrac{2\beta k_{\mathrm{B}} T}{m}\int_0^t\left[\left(\varphi^+(\tau) - \alpha(k_{xx}-k_{yy})\varphi^-(\tau)\right)^2 + \left(2\alpha k_{xy}\varphi^-(\tau)\right)^2\right]d\tau, \\ \left\langle y^2\right\rangle = \left\langle y\right\rangle^2 + \dfrac{2\beta k_{\mathrm{B}} T}{m}\int_0^t\left[\left(\varphi^+(\tau) + \alpha(k_{xx}-k_{yy})\varphi^-(\tau)\right)^2 + \left(2\alpha k_{yx}\varphi^-(\tau)\right)^2\right]d\tau. \end{cases} \tag{S.34}$$

Using similar procedures, we can derive the following:

$$\left\langle xy\right\rangle = \left\langle x\right\rangle\left\langle y\right\rangle + \frac{4\beta k_{\mathrm{B}} T}{m}\int_0^t\left[\alpha^2(k_{xx}-k_{yy})(k_{yx}-k_{xy})\varphi^-(\tau)^2 - \alpha(k_{yx}+k_{xy})\varphi^+(\tau)\varphi^-(\tau)\right]d\tau, \tag{S.35}$$

$$\begin{cases} \left\langle v_x\right\rangle = -[d_1^+ - \alpha(k_{xx}-k_{yy})d_1^-]x_0 + 2\alpha k_{xy}d_1^- y_0 + [d_2^+ - \alpha(k_{xx}-k_{yy})d_2^-]v_{0x} - 2\alpha k_{xy}d_2^- v_{0y}, \\ \left\langle v_y\right\rangle = 2\alpha k_{yx}d_1^- x_0 - [d_1^+ + \alpha(k_{xx}-k_{yy})d_1^-]y_0 - 2\alpha k_{yx}d_2^- v_{0x} + [d_2^+ + \alpha(k_{xx}-k_{yy})d_2^-]v_{0y}, \end{cases} \tag{S.36}$$

and

$$\begin{cases} \left\langle v_x^2 \right\rangle = \left\langle v_x \right\rangle^2 + \dfrac{2\beta k_{\mathrm{B}} T}{m} \int_0^t \left[\left(\phi^+(\tau) - \alpha(k_{xx} - k_{yy}) \phi^-(\tau) \right)^2 + \left(2\alpha k_{xy} \phi^-(\tau) \right)^2 \right] d\tau, \\ \left\langle v_y^2 \right\rangle = \left\langle v_y \right\rangle^2 + \dfrac{2\beta k_{\mathrm{B}} T}{m} \int_0^t \left[\left(\phi^+(\tau) - \alpha(k_{xx} - k_{yy}) \phi^-(\tau) \right)^2 + \left(2\alpha k_{yx} \phi^-(\tau) \right)^2 \right] d\tau, \\ \left\langle v_x v_y \right\rangle = \left\langle v_x \right\rangle \left\langle v_y \right\rangle + \dfrac{4\beta k_{\mathrm{B}} T}{m} \int_0^t \left[\alpha^2 (k_{xx} - k_{yy})(k_{yx} - k_{xy}) \phi^-(\tau)^2 - \alpha(k_{yx} + k_{xy}) \phi^+(\tau) \phi^-(\tau) \right] d\tau, \end{cases}$$

(S.37)

where

$$\begin{cases} d_1^{\pm} = \dfrac{1}{2} \left(\dfrac{\mu_1^+ \mu_1^- \left(e^{\mu_1^+ t} - e^{\mu_1^- t} \right)}{\mu_1^+ - \mu_1^-} \pm \dfrac{\mu_2^+ \mu_2^- \left(e^{\mu_2^+ t} - e^{\mu_2^- t} \right)}{\mu_2^+ - \mu_2^-} \right), \\ d_2^{\pm} = \dfrac{1}{2} \left(\dfrac{\mu_1^+ e^{\mu_1^+ t} - \mu_1^- e^{\mu_1^- t}}{\mu_1^+ - \mu_1^-} \pm \dfrac{\mu_2^+ e^{\mu_2^+ t} - \mu_2^- e^{\mu_2^- t}}{\mu_2^+ - \mu_2^-} \right), \end{cases} \quad \text{(S.38)}$$

and

$$\phi^{\pm}(\tau) = \frac{1}{2} \left(\phi_1(\tau) \pm \phi_2(\tau) \right). \quad \text{(S.39)}$$

After taking $\mathrm{Re}\left(\mu_i^{\pm}\right) < 0$ for i = 1, 2 and $t \to \infty$, we have $b_i^{\pm} \to 0$ and $d_i^{\pm} \to 0$, which in turn give $\langle x \rangle \to 0$, $\langle y \rangle \to 0$, $\langle v_x \rangle \to 0$ and $\langle v_y \rangle \to 0$. Then, Eqs. (S.34), (S.35), and (S.37) become

$$\begin{cases} \left\langle x^2 \right\rangle = \dfrac{2\beta k_{\mathrm{B}} T}{m} \int_0^t \left[\left(\varphi^+(\tau) - \alpha(k_{xx} - k_{yy}) \varphi^-(\tau) \right)^2 + \left(2\alpha k_{xy} \varphi^-(\tau) \right)^2 \right] d\tau, \\ \left\langle y^2 \right\rangle = \dfrac{2\beta k_{\mathrm{B}} T}{m} \int_0^t \left[\left(\varphi^+(\tau) + \alpha(k_{xx} - k_{yy}) \varphi^-(\tau) \right)^2 + \left(2\alpha k_{yx} \varphi^-(\tau) \right)^2 \right] d\tau, \\ \left\langle xy \right\rangle = \dfrac{4\beta k_{\mathrm{B}} T}{m} \int_0^t \left[\alpha^2 (k_{xx} - k_{yy})(k_{yx} - k_{xy}) \varphi^-(\tau)^2 - \alpha(k_{yx} + k_{xy}) \varphi^+(\tau) \varphi^-(\tau) \right] d\tau, \\ \left\langle v_x^2 \right\rangle = \dfrac{2\beta k_{\mathrm{B}} T}{m} \int_0^t \left[\left(\phi^+(\tau) - \alpha(k_{xx} - k_{yy}) \phi^-(\tau) \right)^2 + \left(2\alpha k_{xy} \phi^-(\tau) \right)^2 \right] d\tau, \\ \left\langle v_y^2 \right\rangle = \dfrac{2\beta k_{\mathrm{B}} T}{m} \int_0^t \left[\left(\phi^+(\tau) - \alpha(k_{xx} - k_{yy}) \phi^-(\tau) \right)^2 + \left(2\alpha k_{yx} \phi^-(\tau) \right)^2 \right] d\tau, \\ \left\langle v_x v_y \right\rangle = \dfrac{4\beta k_{\mathrm{B}} T}{m} \int_0^t \left[\alpha^2 (k_{xx} - k_{yy})(k_{yx} - k_{xy}) \phi^-(\tau)^2 - \alpha(k_{yx} + k_{xy}) \phi^+(\tau) \phi^-(\tau) \right] d\tau. \end{cases} \quad \text{(S.40)}$$

By further simplifying, and taking $\mathrm{Re}\left(\mu_i^{\pm}\right)<0$ and $t\to\infty$, the expectation values converge to the steady-state value. Equation (S.40) is simplified to

$$
\begin{cases}
\dfrac{1}{2}\dfrac{k_{xx}+k_{yy}}{2}\left\langle x^2\right\rangle=\left(\dfrac{k_{xy}(k_{xy}-k_{yx})+k_{yy}(k_{xx}+k_{yy})}{4k_{xy}k_{yx}-4k_{xx}k_{yy}}+\dfrac{k_{xy}(-k_{xy}+k_{yx})}{4k_{xy}k_{yx}+(k_{xx}-k_{yy})^2-2(k_{xx}+k_{yy})\gamma^2/m}\right)k_{\mathrm{B}}T, \\
\dfrac{1}{2}\dfrac{k_{xx}+k_{yy}}{2}\left\langle y^2\right\rangle=\left(\dfrac{k_{yx}(-k_{xy}+k_{yx})+k_{xx}(k_{xx}+k_{yy})}{4k_{xy}k_{yx}-4k_{xx}k_{yy}}+\dfrac{k_{yx}(k_{xy}-k_{yx})}{4k_{xy}k_{yx}+(k_{xx}-k_{yy})^2-2(k_{xx}+k_{yy})\gamma^2/m}\right)k_{\mathrm{B}}T, \\
\dfrac{1}{2}\dfrac{k_{xx}+k_{yy}}{2}\left\langle xy\right\rangle=\left(\dfrac{k_{xx}k_{xy}+k_{yx}k_{yy}}{-4k_{xy}k_{yx}+4k_{xx}k_{yy}}+\dfrac{(k_{xy}-k_{yx})(k_{xx}-k_{yy})}{2\left(4k_{xy}k_{yx}+(k_{xx}-k_{yy})^2-2(k_{xx}+k_{yy})\gamma^2/m\right)}\right)k_{\mathrm{B}}T, \\
\dfrac{1}{2}m\left\langle v_x^2\right\rangle=\left(\dfrac{1}{2}+\dfrac{k_{xy}(k_{xy}-k_{yx})}{4k_{xy}k_{yx}+(k_{xx}-k_{yy})^2-2(k_{xx}+k_{yy})\gamma^2/m}\right)k_{\mathrm{B}}T, \\
\dfrac{1}{2}m\left\langle v_y^2\right\rangle=\left(\dfrac{1}{2}+\dfrac{k_{yx}(-k_{xy}+k_{yx})}{4k_{xy}k_{yx}+(k_{xx}-k_{yy})^2-2(k_{xx}+k_{yy})\gamma^2/m}\right)k_{\mathrm{B}}T, \\
\dfrac{1}{2}m\left\langle v_x v_y\right\rangle=\left(\dfrac{-(k_{xy}-k_{yx})(k_{xx}-k_{yy})}{2\left(4k_{xy}k_{yx}+(k_{xx}-k_{yy})^2-2(k_{xx}+k_{yy})\gamma^2/m\right)}\right)k_{\mathrm{B}}T.
\end{cases}
$$

(S.41)

It is possible to orientate the coordinate (e.g. by diagonalizing the symmetric part of the force matrix) such that $k_{xy}=-k_{yx}=g$; then, we have

$$
\begin{cases}
\frac{1}{2}\frac{k_{xx}+k_{yy}}{2}\left\langle x^2\right\rangle = \left(-\frac{2g^2+k_{yy}(k_{xx}+k_{yy})}{4(g^2+k_{xx}k_{yy})}-\frac{2g^2}{-4g^2+(k_{xx}-k_{yy})^2-2(k_{xx}+k_{yy})\gamma^2/m}\right)k_{\mathrm{B}}T, \\
\frac{1}{2}\frac{k_{xx}+k_{yy}}{2}\left\langle y^2\right\rangle = \left(-\frac{2g^2+k_{xx}(k_{xx}+k_{yy})}{4(g^2+k_{xx}k_{yy})}-\frac{2g^2}{-4g^2+(k_{xx}-k_{yy})^2-2(k_{xx}+k_{yy})\gamma^2/m}\right)k_{\mathrm{B}}T, \\
\frac{1}{2}\frac{k_{xx}+k_{yy}}{2}\left\langle xy\right\rangle = \left(\frac{g(k_{xx}-k_{yy})}{4\left(g^2+k_{xx}k_{yy}\right)}+\frac{g(k_{xx}-k_{yy})}{-4g^2+(k_{xx}-k_{yy})^2-2(k_{xx}+k_{yy})\gamma^2/m}\right)k_{\mathrm{B}}T, \\
\frac{1}{2}m\left\langle v_x^2\right\rangle = \left(\frac{1}{2}+\frac{2g^2}{-4g^2+(k_{xx}-k_{yy})^2-2(k_{xx}+k_{yy})\gamma^2/m}\right)k_{\mathrm{B}}T, \\
\frac{1}{2}m\left\langle v_y^2\right\rangle = \left(\frac{1}{2}+\frac{2g^2}{-4g^2+(k_{xx}-k_{yy})^2-2(k_{xx}+k_{yy})\gamma^2/m}\right)k_{\mathrm{B}}T, \\
\frac{1}{2}m\left\langle v_x v_y\right\rangle = \left(\frac{-g(k_{xx}-k_{yy})}{-4g^2+(k_{xx}-k_{yy})^2-2(k_{xx}+k_{yy})\gamma^2/m}\right)k_{\mathrm{B}}T.
\end{cases}
$$

(S.42)

Equation (S.42) defines the NHNE theory for motion confined in a flat 2D plane, which is the major result of this paper.

We stress that the problem we resolved is a multi-dimensional non-Hermitian problem within the framework of the Langevin equation. In contrast, owing to the Hermiticity, the 3 cartesian directions are independent in Ref. [i] therefore the problem reduced to a single dimension one.

Supplementary Note 2: Derivation of the NHNE theory in three-dimensional (3D) space

The derivation outlined in Supplementary Note 1 can be generalized for a Brownian particle in 3D space and characterized by a general 3 × 3 force matrix $\overleftrightarrow{\mathbf{K}}$. By applying $b_i^{\pm} \to 0$ and $d_i^{\pm} \to 0$ and starting from Eq. (S.19), the displacement is given by

$$\mathbf{r} = \int_0^t \overleftrightarrow{\mathbf{\Lambda}} \begin{bmatrix} \varphi_1(\tau) & 0 & 0 \\ 0 & \varphi_2(\tau) & 0 \\ 0 & 0 & \varphi_3(\tau) \end{bmatrix} \overleftrightarrow{\mathbf{\Lambda}}^{-1} \frac{1}{m} \begin{bmatrix} A_1(\tau) \\ A_2(\tau) \\ A_3(\tau) \end{bmatrix} d\tau \,, \tag{S.43}$$

and the velocity is given by

$$\mathbf{v} = \int_0^t \overleftrightarrow{\mathbf{\Lambda}} \begin{bmatrix} \phi_1(\tau) & 0 & 0 \\ 0 & \phi_2(\tau) & 0 \\ 0 & 0 & \phi_3(\tau) \end{bmatrix} \overleftrightarrow{\mathbf{\Lambda}}^{-1} \frac{1}{m} \begin{bmatrix} A_1(\tau) \\ A_2(\tau) \\ A_3(\tau) \end{bmatrix} d\tau \,. \tag{S.44}$$

By following the derivation in Supplementary Note 1, without significant extra difficulty, we eventually obtain the ensemble averages in three dimensions, namely:

$$\begin{aligned} \langle r_i r_j \rangle &= \frac{2\gamma k_B T}{m^2} \sum_{n=1}^{3} \sum_{l=1}^{3} \sum_{m=1}^{3} \left[\overleftrightarrow{\mathbf{\Lambda}}_{il} (\overleftrightarrow{\mathbf{\Lambda}}^{-1})_{mn} \right] \left[\overleftrightarrow{\mathbf{\Lambda}}_{jm} (\overleftrightarrow{\mathbf{\Lambda}}^{-1})_{ln} \right] \overleftrightarrow{\mathbf{M}}^{\varphi}_{ml} \,, \\ \langle v_i v_j \rangle &= \frac{2\gamma k_B T}{m^2} \sum_{n=1}^{3} \sum_{l=1}^{3} \sum_{m=1}^{3} \left[\overleftrightarrow{\mathbf{\Lambda}}_{il} (\overleftrightarrow{\mathbf{\Lambda}}^{-1})_{mn} \right] \left[\overleftrightarrow{\mathbf{\Lambda}}_{jm} (\overleftrightarrow{\mathbf{\Lambda}}^{-1})_{ln} \right] \overleftrightarrow{\mathbf{M}}^{\phi}_{ml} \,, \end{aligned} \tag{S.45}$$

where the columns of $\overleftrightarrow{\mathbf{\Lambda}}$ are the right eigenvectors for $-\overleftrightarrow{\mathbf{K}}/m$, $\overleftrightarrow{\mathbf{\Lambda}}_{il}$ denotes the matrix element at the ith row and l^{th} column, and

$$\begin{cases} \overleftrightarrow{\mathbf{M}}^{\varphi} = \int_0^t \begin{bmatrix} \varphi_1(\tau) \\ \varphi_2(\tau) \\ \varphi_3(\tau) \end{bmatrix} \otimes \begin{bmatrix} \varphi_1(\tau) & \varphi_2(\tau) & \varphi_3(\tau) \end{bmatrix} d\tau, \\ \overleftrightarrow{\mathbf{M}}^{\phi} = \int_0^t \begin{bmatrix} \phi_1(\tau) \\ \phi_2(\tau) \\ \phi_3(\tau) \end{bmatrix} \otimes \begin{bmatrix} \phi_1(\tau) & \phi_2(\tau) & \phi_3(\tau) \end{bmatrix} d\tau, \end{cases} \tag{S.46}$$

where $\varphi_i(\tau)$ and $\phi_i(\tau)$ are given by Eqs. (S.13) and (S.14), respectively, and $\otimes$ denotes the Kronecker product. The system is stable only if $\mathrm{Re}\left(\mu_i^{\pm}\right) < 0$ for all *i*. With this assumed, and by taking the limit $t \to \infty$, Eq. (S.46) is simplified to

$$\begin{cases} \overleftrightarrow{\mathbf{M}}_{ml}^{\varphi} = -\dfrac{\mu_m^+ + \mu_m^- + \mu_l^+ + \mu_l^-}{\left(\mu_m^+ + \mu_l^+\right)\left(\mu_m^- + \mu_l^+\right)\left(\mu_m^+ + \mu_l^-\right)\left(\mu_m^- + \mu_l^-\right)}, \\ \overleftrightarrow{\mathbf{M}}_{ml}^{\phi} = -\dfrac{\mu_m^- \mu_l^+ \left(\mu_m^+ + \mu_l^-\right) + \mu_m^+ \mu_l^- \left(\mu_m^- + \mu_l^+\right)}{\left(\mu_m^+ + \mu_l^+\right)\left(\mu_m^- + \mu_l^+\right)\left(\mu_m^+ + \mu_l^-\right)\left(\mu_m^- + \mu_l^-\right)}, \end{cases} \tag{S.47}$$

where $\mu_i^{\pm}$ is defined in Eq. (S.15).

Supplementary Note 3: Virial theorem for a non-Hermitian force field

The virial theorem [iii] states that for a trapped Brownian particle, the averaged kinetic energy for the ith Cartesian degree of freedom is given by

$$\langle T_i \rangle = \frac{1}{2} m \langle v_i^2 \rangle = -\frac{1}{2} \langle F_i \cdot r_i \rangle, \tag{S.48}$$

where $\mathbf{F}(\mathbf{r} = (x, y, z)) = (F_x, F_y, F_z)$ is the trapping force, and $\mathbf{r}$ is the displacement from the equilibrium. The total kinetic energy is given by

$$\langle T_x + T_y + T_z \rangle = \frac{1}{2} m \langle v_x^2 + v_y^2 + v_z^2 \rangle = -\frac{1}{2} \sum_{i=1}^{3} \langle F_i \cdot r_i \rangle. \tag{S.49}$$

where v_i, F_i and r_i are the ith Cartesian components of $\mathbf{v}$, $\mathbf{F}$ and $\mathbf{r}$, respectively.

Unlike the ET, the virial theorem of Eq. (S.48) applies to a non-Hermitian trapping force ($\overleftrightarrow{\mathbf{K}} \neq \overleftrightarrow{\mathbf{K}}^\dagger$), as expected. To illustrate this explicitly, we substitute $\mathbf{F} = \overleftrightarrow{\mathbf{K}}_{\text{2D}} \cdot \mathbf{r}$ into Eq. (S.48), where $\overleftrightarrow{\mathbf{K}}_{\text{2D}} = \begin{bmatrix} k_{xx} & k_{xy} \\ k_{yx} & k_{yy} \end{bmatrix}$, to obtain

$$\begin{aligned} \frac{1}{2} m \langle v_x^2 \rangle &= -\frac{1}{2} k_{xx} \langle x^2 \rangle - \frac{1}{2} k_{xy} \langle xy \rangle, \\ \frac{1}{2} m \langle v_y^2 \rangle &= -\frac{1}{2} k_{yy} \langle y^2 \rangle - \frac{1}{2} k_{yx} \langle xy \rangle. \end{aligned} \tag{S.50}$$

Equation (S.50) can be fulfilled by using $\langle x^2 \rangle$, $\langle y^2 \rangle$, $\langle v_x^2 \rangle$, $\langle v_y^2 \rangle$ and $\langle xy \rangle$ from Eq. (S.41)

Interestingly, both the kinetic energy and "potential" energy associated with a single degree of freedom depend on the orientation of the coordinate system, whereas the total kinetic energy remains constant. This can be seen by comparing the two cases shown in Supplementary Fig. 1a and 1d. In the former, $\overleftrightarrow{\mathbf{K}}_{\text{2D}} = \begin{bmatrix} k_{xx} & g \\ -g & k_{yy} \end{bmatrix}$ is unrotated ($\theta_{\text{rot}} = 0°$), whereas in the latter, $\overleftrightarrow{\mathbf{K}}'_{\text{2D}} = \overleftrightarrow{\mathbf{R}}(\theta_{\text{rot}}) \overleftrightarrow{\mathbf{K}}_{\text{2D}} \overleftrightarrow{\mathbf{R}}(\theta_{\text{rot}})^{-1} = \begin{bmatrix} k'_{xx} & k'_{xy} \\ k'_{yx} & k'_{yy} \end{bmatrix}$ is rotated ($\theta_{\text{rot}} = 45°$). Here, $\overleftrightarrow{\mathbf{R}}(\theta_{\text{rot}}) = \begin{bmatrix} \cos\theta_{\text{rot}} & \sin\theta_{\text{rot}} \\ -\sin\theta_{\text{rot}} & \cos\theta_{\text{rot}} \end{bmatrix}$ represents the rotational matrix. The average kinetic and "potential"

energies versus incident polarization are plotted in Supplementary Fig. 1b–c and Supplementary Fig. 1e–f, respectively. The kinetic energies (left column) are in complete agreement with the "potential" energy (right column), confirming the validity of Eq. (S.50). For some configurations, the average energies for certain degrees of freedom can even be less than $k_B T/2$ (see Supplementary Fig. 1e and 1f). In all cases, the total average kinetic energy remains constant regardless of the choice of coordinates, as expected. To prove this explicitly, for an arbitrary θ_{rot}, the force matrix has the form

$$\overleftrightarrow{\mathbf{K}}'_{\text{2D}} = \overleftrightarrow{\mathbf{R}}(\theta_{\text{rot}})\overleftrightarrow{\mathbf{K}}_{\text{2D}}\overleftrightarrow{\mathbf{R}}(\theta_{\text{rot}})^{-1} = \begin{bmatrix} k'_{xx} & k'_{xy} \\ k'_{yx} & k'_{yy} \end{bmatrix}$$
$$= \begin{bmatrix} \frac{1}{2}\left(k_{xx}+k_{yy}+(k_{xx}-k_{yy})\cos 2\theta_{\text{rot}}\right) & g-(k_{xx}-k_{yy})\sin\theta_{\text{rot}}\cos\theta_{\text{rot}} \\ -g-(k_{xx}-k_{yy})\sin\theta_{\text{rot}}\cos\theta_{\text{rot}} & \frac{1}{2}\left(k_{xx}+k_{yy}-(k_{xx}-k_{yy})\cos 2\theta_{\text{rot}}\right) \end{bmatrix}. \quad \text{(S.51)}$$

The total kinetic energy can be calculated by Eq. (S.50):

$$\begin{aligned} &\frac{1}{2}m\left\langle v_x'^2\right\rangle + \frac{1}{2}m\left\langle v_y'^2\right\rangle \\ &= -\frac{1}{2}k'_{xx}\left\langle x'^2\right\rangle - \frac{1}{2}k'_{xy}\left\langle x'y'\right\rangle - \frac{1}{2}k'_{yy}\left\langle y'^2\right\rangle - \frac{1}{2}k'_{yx}\left\langle x'y'\right\rangle \\ &= \left(1+\frac{k'_{xy}(k'_{xy}-k'_{yx})+k'_{yx}(-k'_{xy}+k'_{yx})}{4k'_{xy}k'_{yx}+(k'_{xx}-k'_{yy})^2-2(k'_{xx}+k'_{yy})\gamma^2/m}\right)k_B T, \\ &= \left(1+\frac{4g^2}{-4g^2+(k_{xx}-k_{yy})^2-2(k_{xx}+k_{yy})\gamma^2/m}\right)k_B T, \end{aligned} \quad \text{(S.52)}$$

where the force constants are substituted by the corresponding components of $\overleftrightarrow{\mathbf{K}}'_{\text{2D}}$ in Eq. (S.51), and the averaged quantities in $\langle\cdots\rangle$ are those in Eq. (S.41). Equation (S.52) shows that the total kinetic energy is independent of θ_{rot}.

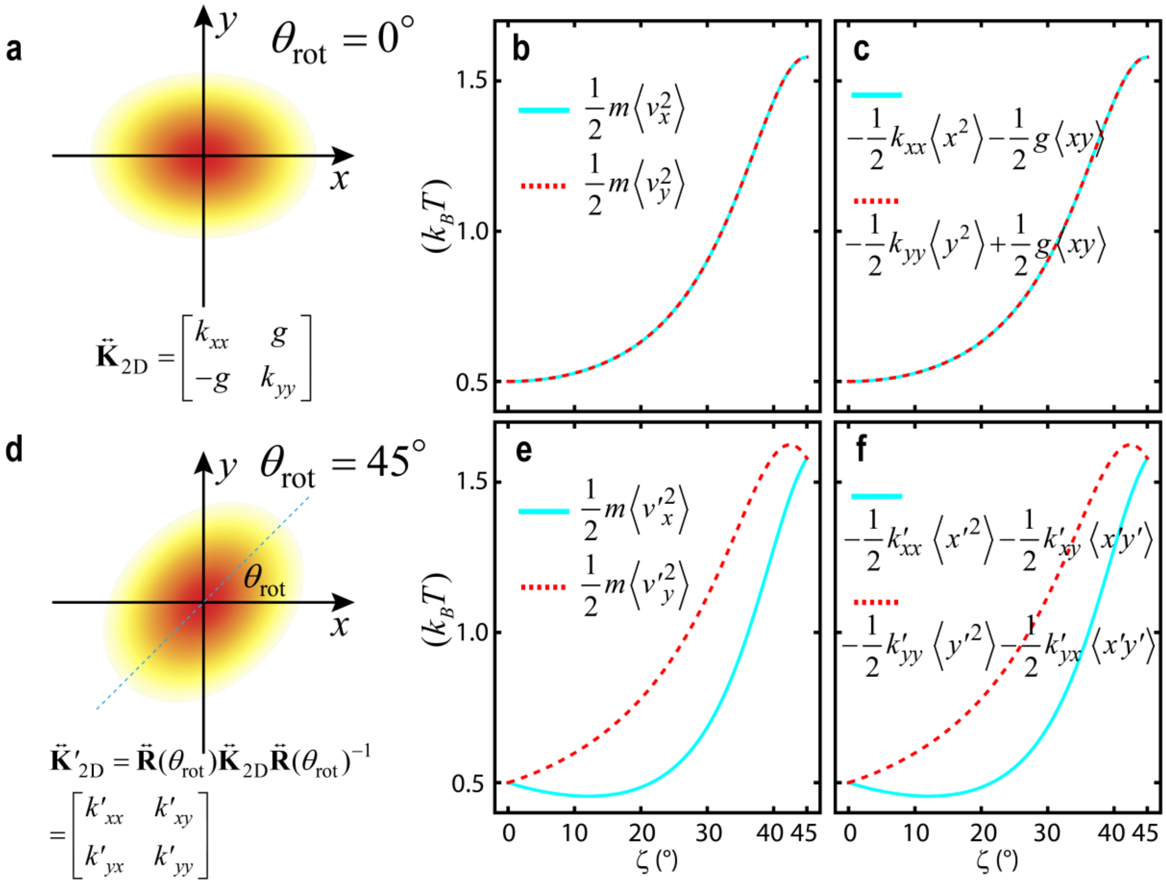

Supplementary Fig. 1| Coordinate dependency of the energy associated with a single degree of freedom. Intensity of the incident beam at (a) $\theta_{rot} = 0°$ and (d) $\theta_{rot} = 45°$. (b) and (c) show the kinetic energies and "potential" energies for $\theta_{rot} = 0°$, whereas (e) and (f) show the kinetic energies and "potential" energies for $\theta_{rot} = 45°$. The agreement between (b) and (c) ((e) and (f)) verifies the virial theorem. The disagreement between (b–c) and (e–f) illustrates the coordinate dependency.

Supplementary Note 4: Analytical expressions for the rate at which work is done by the forces plotted in Fig. 2 in the main text

Using the averaged quantities derived in Supplementary Note 1, we can represent the rate at which work is done by the damping force

$$\begin{aligned}
&\langle \gamma \mathbf{v}\cdot\mathbf{v}\rangle = \langle \gamma v_x^2\rangle + \langle \gamma v_y^2\rangle \\
&= \left(\begin{array}{l} \dfrac{1}{2} + \dfrac{k_{xy}(k_{xy}-k_{yx})}{4k_{xy}k_{yx}+(k_{xx}-k_{yy})^2-2(k_{xx}+k_{yy})\gamma^2/m} \\ +\dfrac{1}{2} + \dfrac{k_{yx}(-k_{xy}+k_{yx})}{4k_{xy}k_{yx}+(k_{xx}-k_{yy})^2-2(k_{xx}+k_{yy})\gamma^2/m} \end{array} \right) \frac{2\gamma k_{\mathrm{B}}T}{m}, \\
&= \left(1 + \frac{(k_{xy}-k_{yx})^2}{4k_{xy}k_{yx}+(k_{xx}-k_{yy})^2-2(k_{xx}+k_{yy})\gamma^2/m} \right) \frac{2\gamma k_{\mathrm{B}}T}{m},
\end{aligned} \tag{S.53}$$

by the random force

$$\langle \mathbf{A}(t)\cdot\mathbf{v}\rangle = \langle A_x(t)v_x\rangle + \langle A_y(t)v_y\rangle = \frac{\gamma k_{\mathrm{B}}T}{m} + \frac{\gamma k_{\mathrm{B}}T}{m} = \frac{2\gamma k_{\mathrm{B}}T}{m}, \tag{S.54}$$

and by the non-Hermitian force

$$\begin{aligned}
&\langle \mathbf{F}(\mathbf{r})\cdot\mathbf{v}\rangle = k_{xx}\langle x v_x\rangle + k_{xy}\langle x v_y\rangle + k_{yx}\langle y v_x\rangle + k_{yy}\langle y v_y\rangle \\
&= 0 + k_{xy}\frac{2(k_{xy}-k_{yx})\gamma/m}{4k_{xy}k_{yx}+(k_{xx}-k_{yy})^2-2(k_{xx}+k_{yy})\gamma^2/m} k_{\mathrm{B}}T \\
&+ k_{yx}\frac{2(-k_{xy}+k_{yx})\gamma/m}{4k_{xy}k_{yx}+(k_{xx}-k_{yy})^2-2(k_{xx}+k_{yy})\gamma^2/m} k_{\mathrm{B}}T + 0, \\
&= \frac{(k_{xy}-k_{yx})^2}{4k_{xy}k_{yx}+(k_{xx}-k_{yy})^2-2(k_{xx}+k_{yy})\gamma^2/m} \frac{2\gamma k_{\mathrm{B}}T}{m}.
\end{aligned} \tag{S.55}$$

It can be explicitly verified that

$$\langle \mathbf{F}(\mathbf{r})\cdot\mathbf{v}\rangle + \langle \mathbf{A}(t)\cdot\mathbf{v}\rangle - \langle \gamma \mathbf{v}\cdot\mathbf{v}\rangle = 0, \tag{S.56}$$

which is required by energy conservation.

Supplementary Note 5: Statistics of a particle trapped by a non-Hermitian force field

In general, the trap stiffnesses k_{xx} and k_{yy} are different. Thus, a trapped particle undergoing Brownian fluctuation has an anisotropic spatial probability distribution [iv],

$$P(x,y) \propto e^{\left[-\frac{1}{2}\mathbf{r}^{\mathrm{T}}\left\langle \mathbf{r}\mathbf{r}^{\mathrm{T}}\right\rangle^{-1}\mathbf{r}\right]}, \tag{S.57}$$

whose profile is dictated by the unit ellipse defined by

$$\mathbf{r}^{\mathrm{T}} \cdot \left\langle \mathbf{r}\mathbf{r}^{\mathrm{T}}\right\rangle^{-1} \cdot \mathbf{r} = 1. \tag{S.58}$$

Equation (S.58) can be expressed in polar coordinates (r,θ) as

$$r = \sqrt{\frac{\left\langle x^2\right\rangle\left\langle y^2\right\rangle - \left\langle xy\right\rangle^2}{\left\langle x^2\right\rangle \sin^2\theta - 2\left\langle xy\right\rangle \sin\theta\cos\theta + \left\langle y^2\right\rangle\cos^2\theta}}. \tag{S.59}$$

From Eq. (S.59), the angle ($\theta_{ellipse}$) between the major axis of the ellipse and the *x*-axis is

$$\tan 2\theta_{ellipse} = \frac{2\left\langle xy\right\rangle}{\left\langle x^2\right\rangle - \left\langle y^2\right\rangle}. \tag{S.60}$$

By substituting $\left\langle x^2\right\rangle$, $\left\langle y^2\right\rangle$, and $\left\langle xy\right\rangle$ from Eq. (S.42) into Eq. (S.60), we obtain:

$$\tan 2\theta_{ellipse} = \frac{2g(k_{xx} + k_{yy} - 2\gamma^2/m)}{-4g^2 + (k_{xx} - k_{yy})^2 - 2(k_{xx} + k_{yy})\gamma^2/m}. \tag{S.61}$$

Evidently, $\theta_{ellipse} \to 0$ as $g \to 0$, thus the ellipse is misaligned with the axis of the restoring forces (*x* and *y* axis) if and only if *g* is non-zero. Supplementary Fig. 2a plots the particle trajectories, from which the orientation of the ellipses can be inferred. The parameters are identical to those in Fig. 1 of the main text. The rotation of the probability distributions with increasing ζ (or *g*) is evident in Supplementary Fig. 2a. A plot of $\theta_{ellipse}$ versus ζ is shown in Supplementary Fig. 2b, with the analytical results calculated using Eq. (S.61) and the numerical results calculated using Eq. (S.60). Remarkable agreement is observed.

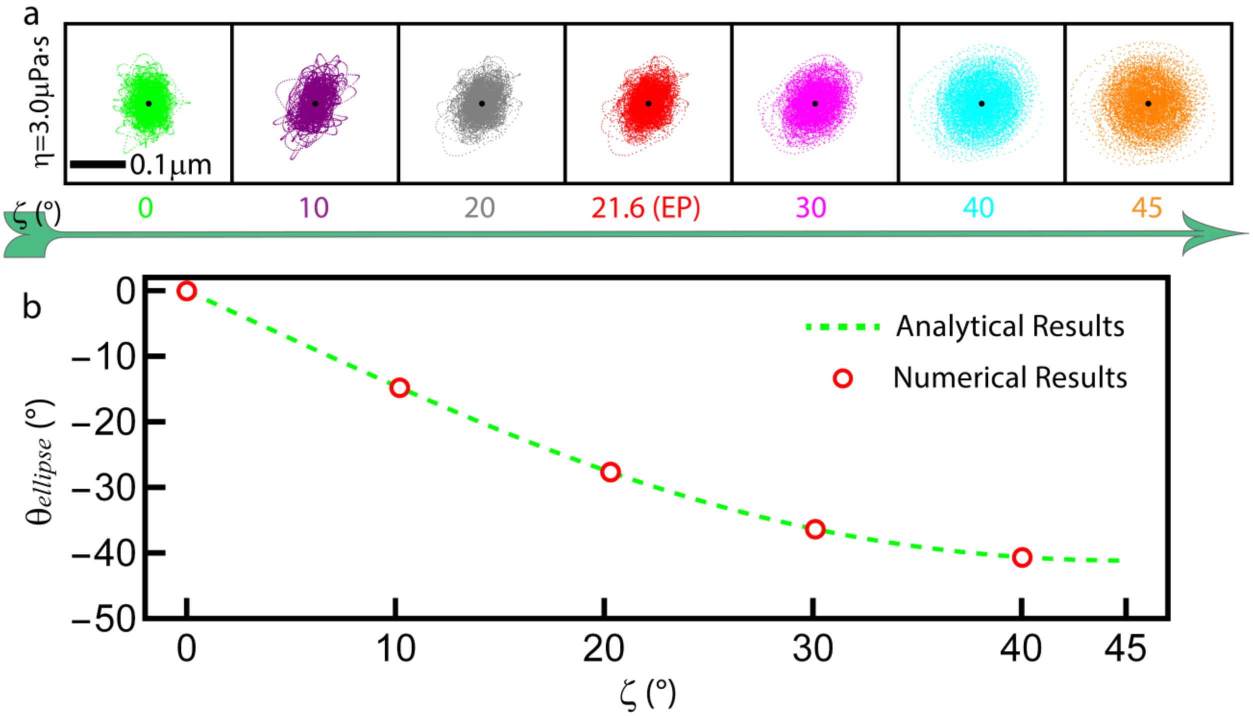

Supplementary Fig. 2| Trajectories of an optically trapped Brownian particle versus ζ (polarization) under the same conditions as depicted Fig. 1 in the main text. (a) Trajectories at various ζ, where the duration of the trajectories is 0.01 s. (b) Orientation of the trajectories ($\theta_{ellipse}$) versus ζ.

Supplementary Note 6: Phase diagram for the NHNE theory in single-particle trapping

Consider a particle of radius a and mass m immersed in a fluid, with the particle trapped by an elliptically polarized Gaussian beam. The ambient friction coefficient is $\gamma = 6\pi\eta a$, while the non-Hermitian optical force is $\mathbf{F} = \overleftrightarrow{\mathbf{K}} \cdot \mathbf{r} = P\overleftrightarrow{\mathbf{K}}_0 \cdot \mathbf{r}$, where P denotes the beam power, and $\overleftrightarrow{\mathbf{K}}_0$ is the force matrix when the power is 1.0 W. According to the K_0^i, the ith eigenvalue of $\overleftrightarrow{\mathbf{K}}_0$, we can categorize the stability as in the following Supplementary Table 1 [ii, v], where $\gamma_{\text{cri}} = 6\pi\eta_{\text{cri}} a = \sqrt{mP}\dfrac{|\text{Im}(K_0^i)|}{\sqrt{|\text{Re}(K_0^i)|}}$ is the critical friction coefficient.

Any of $\text{Re}(K_0^i) > 0$	All of $\text{Re}(K_0^i) < 0$	
Unstable (pink)	$\gamma < \gamma_{\text{cri}}$	$\gamma \geq \gamma_{\text{cri}}$
	Unstable (red)	Stable (blue)

Supplementary Table 1| Stability versus K_0^i. $\gamma_{\text{cri}} = 6\pi\eta_{\text{cri}} a = \sqrt{mP}\dfrac{|\text{Im}(K_0^i)|}{\sqrt{|\text{Re}(K_0^i)|}}$ is the critical friction coefficient.

The stability over the phase space for a fixed damping coefficient γ and power P is depicted in Supplementary Fig. 3.

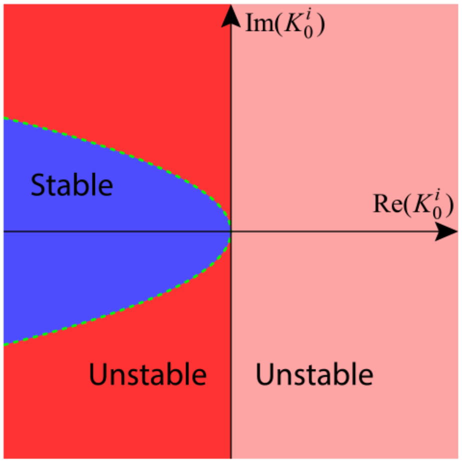

Supplementary Fig. 3| Stability versus K_0^i. Stable region: blue. Unstable region: red and pink. Green dashed line: $\gamma = \gamma_{\mathrm{cri}}$.

Supplementary Fig. 4a (trapping potential) and Supplementary Fig. 4b (saddle potential) display the difference between $\frac{1}{2}m\langle v_x^2\rangle$ and $\frac{1}{2}k_{\mathrm{B}}T$ versus polarizations (ζ) and viscosities (η). The blank regions in the diagrams are unstable regions. For instance, when η = 0, the particle becomes unstable after crossing the exceptional point (EP) in the phase space, marked by red arrows, in both Supplementary Fig. 4a and 4b. These instabilities are caused by insufficient frictional damping ($\gamma < \gamma_{\mathrm{cri}}$). Moreover, owing to the repulsion mode ($K_+ > 0$), the particle is unstable before the neutral point (NP), marked by a blue arrow, for all of the η in Supplementary Fig. 4b.

The phase diagrams of $\frac{1}{2}m\langle v_x^2\rangle - \frac{1}{2}k_{\mathrm{B}}T$ versus polarizations (ζ) and power (P) for trapping potential are also presented in Supplementary Fig. 4c (for η = 3.0 μPa·s) and Supplementary Fig. 4d (for η = 18.4 μPa·s). The particle is always stable when K_i is real on the left-hand side of the EP. However, on the right-hand side of the EP, where K_i is complex, the viscosity η (ambient damping) may be insufficient to stabilize the particle, especially when the P is large (as this causes the critical friction coefficient γ_{cri} to be too large to be reached by ambient damping).

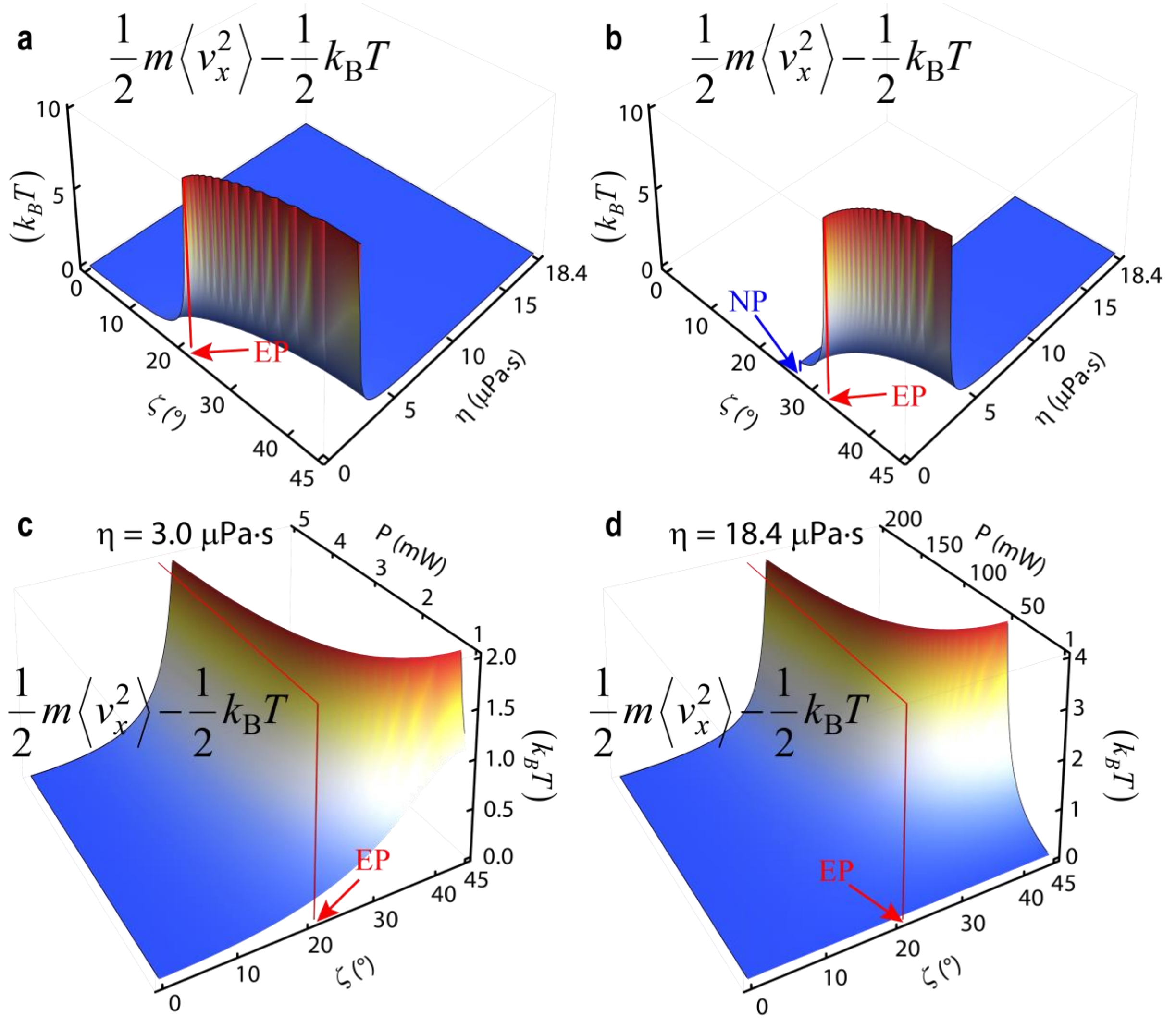

Supplementary Fig. 4| $\frac{1}{2}m\langle v_x^2\rangle - \frac{1}{2}k_BT$ **for an optically trapped Brownian particle versus polarization (*ζ*) and viscosity (*η*).** Panel (a) and (b) represent the trapping potential and saddle potential, respectively, corresponding to Fig. 1 and Fig. 3 in the main text. Panels (c) and (d) represent the trapping potential (Fig. 1 in the main text) with η = 3.0 μPa·s and η = 18.4 μPa·s, respectively. Red and blue arrows mark the exceptional point (EP) and neutral point (NP), respectively.

Supplementary Note 7: The NHNE theory for *N* Brownian particles

Hydrodynamic interactions are hydrodynamic couplings between particles immersed in a fluid and arise because the motion of a particle in a fluid generates a flow field that affects the motion of its neighboring particles. These interactions are determined by the properties of the fluid and by the geometry and motion of the particles. Thus, they can be rather complicated, and depend on factors such as the size, shape, and orientation of the particles, the viscosity and density of the fluid, and the relative velocities of the particles. The influence of hydrodynamic interactions is significant in various systems, such as colloidal suspensions and polymer solutions, and in the microscopic movements of proteins.

By taking hydrodynamic interactions into account, it can be determined that the dynamics of *N* spherical Brownian particles, each with a mass *m*, are governed by a multi-particle version of the Langevin stochastic differential equation [i,vi], that is

$$m\frac{d^2\mathbf{r}}{dt^2} = \mathbf{F}(\mathbf{r}) - \overleftrightarrow{\mathbf{H}}(\mathbf{r})^{-1}\frac{d\mathbf{r}}{dt} + \mathbf{A}(t) \tag{S.62}$$

where $\mathbf{F}(\mathbf{r}) = \left(F_{x,1}, F_{y,1}, F_{z,1}, \cdots, F_{x,N}, F_{y,N}, F_{z,N}\right)$ is the external force (e.g., optical force, acoustic force, or similar) exerted on the N particles, $\mathbf{r} = \left(x_1, y_1, z_1, \cdots, x_N, y_N, z_N\right)$ denotes the particle positions, and $\mathbf{A}(t) = \left(A_{x,1}(t), A_{y,1}(t), A_{z,1}(t), \cdots, A_{x,N}(t), A_{y,N}(t), A_{z,N}(t)\right)$ is the Gaussian-distributed random force due to Brownian fluctuations, with correlations of

$$\left\langle A_i(t)A_j(t')\right\rangle = 2\left(\overleftrightarrow{\mathbf{H}}(\mathbf{r})^{-1}\right)_{ij} k_{\mathrm{B}}T\delta(t-t') \,. \tag{S.63}$$

Here, $A_i(t)$ is the ith component of $\mathbf{A}(t)$, $\left(\overleftrightarrow{\mathbf{H}}(\mathbf{r})^{-1}\right)_{ij}$ denotes the component at the ith row and j^{th} column of $\overleftrightarrow{\mathbf{H}}(\mathbf{r})^{-1}$, and $\overleftrightarrow{\mathbf{H}}(\mathbf{r})$ is the mobility matrix, also known as the Oseen tensor, which characterizes the way in which particles interact with the fluid in their environment (i.e., their hydrodynamic interactions). For N Brownian particles, $\overleftrightarrow{\mathbf{H}}(\mathbf{r})$ is partitioned into $N \times N$ blocks, and each block is a 3 × 3 matrix that is denoted by $\left(\overleftrightarrow{\mathbf{H}}(\mathbf{r})\right)_{3\times3}^{mn}$ and is given by [vi]

$$\left(\overleftrightarrow{\mathbf{H}}(\mathbf{r})\right)_{3\times3}^{mn}=\begin{cases}\dfrac{\overleftrightarrow{\mathbf{I}}_{3\times3}}{\gamma}, & m=n,\\ \dfrac{1}{\gamma}\dfrac{3a}{4R_{mn}}\left(\overleftrightarrow{\mathbf{I}}_{3\times3}+\hat{\mathbf{R}}_{mn}\hat{\mathbf{R}}_{mn}\right), & m\neq n,\end{cases} \tag{S.64}$$

where m and n are the particle indices, $\overleftrightarrow{\mathbf{I}}_{3\times3}$ denotes the 3 × 3 identity matrix, R_{mn} is the distance between the mth and n^{th} particles, $\hat{\mathbf{R}}_{mn}$ is a unit vector in the direction of $\mathbf{R}_{mn}=\mathbf{r}_n-\mathbf{r}_m$ with $\mathbf{r}_m=\left(x_m,y_m,z_m\right)$ being the position of the mth particle, and $\gamma=6\pi\eta a$ is the friction coefficient of a sphere with radius a in a fluid with viscosity η. From Eq. (S.64), it is observed that the hydrodynamic interaction is inversely proportional to the distance between two particles (R_{mn}), and can be neglected if R_{mn} is large or a is small.

Equation (S.62) is rather difficult to solve analytically. However, for small particles or a large separation, we may ignore the hydrodynamic interactions and assume that the random forces acting on the N Brownian particles are completely random. Then, Eq. (S.64) reduces to

$$m\frac{d^2\mathbf{r}}{dt^2}=\mathbf{F}(\mathbf{r})-\gamma\frac{d\mathbf{r}}{dt}+\mathbf{A}(t), \tag{S.65}$$

where the correlations of the Brownian fluctuations are

$$\left\langle A_i(t)A_j(t')\right\rangle=2\gamma k_{\mathrm{B}}T\delta_{i,j}\delta(t-t'). \tag{S.66}$$

We can then analytically determine the approximate NHNE theory for N particles at equilibrium that are coupled by a non-Hermitian binding force:

$$\begin{aligned}\left\langle r_ir_j\right\rangle&=\frac{2\gamma k_{\mathrm{B}}T}{m^2}\sum_{n=1}^{3N}\sum_{l=1}^{3N}\sum_{m=1}^{3N}\left[\overleftrightarrow{\mathbf{\Lambda}}_{il}(\overleftrightarrow{\mathbf{\Lambda}}^{-1})_{mn}\right]\left[\overleftrightarrow{\mathbf{\Lambda}}_{jm}(\overleftrightarrow{\mathbf{\Lambda}}^{-1})_{ln}\right]\overleftrightarrow{\mathbf{M}}_{ml}^{\varphi},\\ \left\langle v_iv_j\right\rangle&=\frac{2\gamma k_{\mathrm{B}}T}{m^2}\sum_{n=1}^{3N}\sum_{l=1}^{3N}\sum_{m=1}^{3N}\left[\overleftrightarrow{\mathbf{\Lambda}}_{il}(\overleftrightarrow{\mathbf{\Lambda}}^{-1})_{mn}\right]\left[\overleftrightarrow{\mathbf{\Lambda}}_{jm}(\overleftrightarrow{\mathbf{\Lambda}}^{-1})_{ln}\right]\overleftrightarrow{\mathbf{M}}_{ml}^{\phi}.\end{aligned} \tag{S.67}$$

While the approximate analytical formula in Eq. (S.67) excludes hydrodynamic interactions, numerical Verlet simulations that take hydrodynamic interaction into account can also be performed. Figure 5 in the main text shows these simulations. By examining Eq. (S.64) and Fig. 5, it can be seen that the approximate multi-particle NHNE theory (Eq. (S.67)) is semi-qualitatively

applicable in scenarios with weak hydrodynamic interactions, such as those with large particle spacings (R_{mn}) or small particle radii (a).

Now, we examine optical binding caused by three z-polarized plane waves, each with an intensity of I_0, propagating on the xy-plane. The wave vectors of the waves form an equilateral triangle, and their interference produces a triangular lattice of high-intensity spots. In the inset of Supplementary Fig. 5b, we show N = 19 particles that are trapped by the lattice of high-intensity spots into a small triangular lattice. In addition to the particles' being trapped by the incident light, the light is also scattered and thus modifies the spatial light distribution, couples the particles, and slightly perturbs the equilibrium positions. This phenomenon is known as optical binding [vii].

The average kinetic energies for each Cartesian degree of freedom ($\frac{1}{2}m\langle v_i^2 \rangle$, i = 1 to 57 (= 3N)) calculated by Eq. (S.67) are plotted in Supplementary Fig. 5a–c as red lines, where I_0 varies from 1 to 100 mW/μm^2. According to the ET, the average kinetic energy is $k_B T/2$ per degree of freedom. However, this is not the case, and the kinetic energies are not equipartitioned. As shown in Supplementary Fig. 5a, at a viscosity of η = 1.0 μPa·s, the energy pumped into the system by light exceeds the energy dissipation at I_0 = 32.2 mW/μm^2. From this point onward, the kinetic energy accumulates and diverges with time, indicating instability. At an increased viscosity of η = 3.0 μPa·s, as shown in Supplementary Fig. 5b, the dissipation is sufficient to remove the energy pumped into the system by light for the intensity range concerned. The average kinetic energy generally increases with I_0 but remains finite, and the cluster is stable. In principle, the average kinetic energy still diverges if I_0 is increased beyond the critical point at $6\pi\eta a = \sqrt{m\mathrm{I}_0}\frac{\left|\mathrm{Im}(K_0^i)\right|}{\sqrt{\left|\mathrm{Re}(K_0^i)\right|}}$ for any mode i, where K_0^i denotes the ith eigenvalue of $\overleftrightarrow{\mathbf{K}}_0$. In Supplementary Fig. 5c, at a viscosity of η = 18.4 μPa·s (air), the situation is similar to that in Supplementary Fig. 5b, except that the average kinetic energy increases by less than 1%, whereas I_0 increases from 1 to 100 mW/μm^2. Supplementary Fig. 5 is expected to have 3N = 57 modes for N = 19 spheres. However, due to degeneracies resulting from rotational and mirror symmetries, only 29 lines are presented.

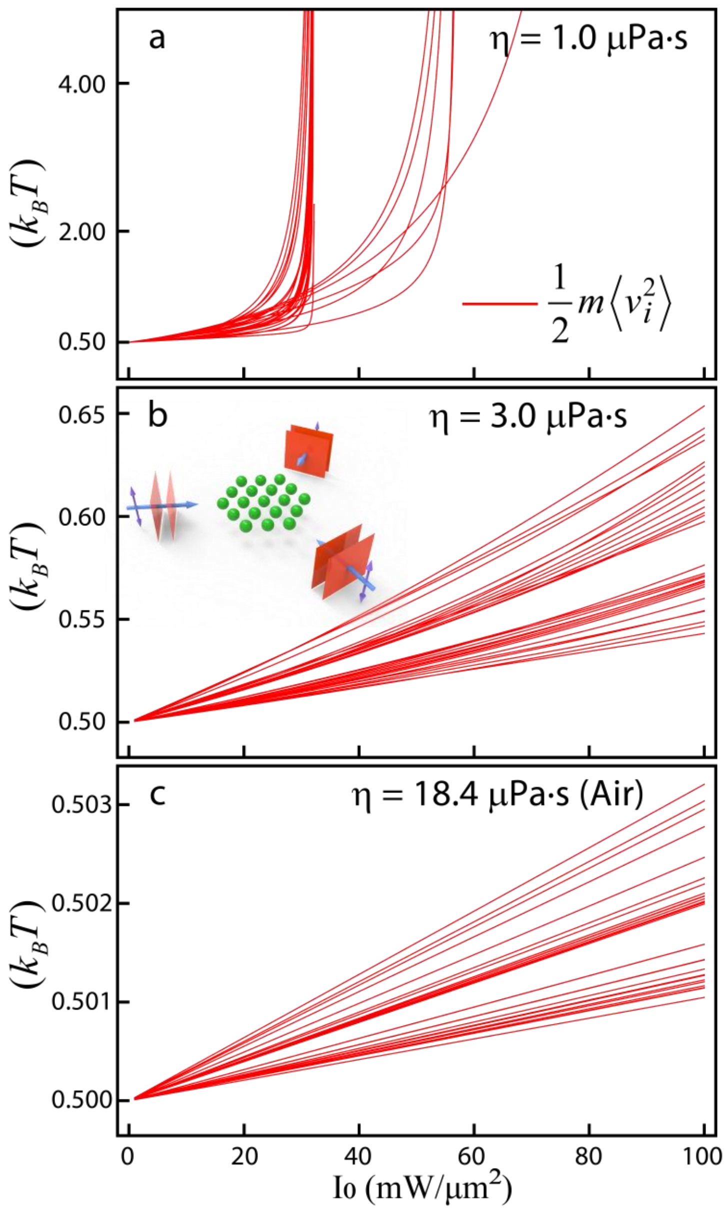

Supplementary Fig. 5| Approximate multi-particle NHNE theory applied to N = 19 spheres (triangular lattice). The 19 spheres, each with a radius a = 0.2 μm and a refractive index n = 1.1, are optically trapped and bound at the high-intensity spots produced by the three linearly polarized plane waves (λ = 1.064 μm) in a low vacuum or in air, as illustrated in the inset in (b). Each plane wave has an intensity of I_0. As I_0 varies from 1 to 100 mW/μm^2, the kinetic energies $\frac{1}{2}m\left\langle v_i^2 \right\rangle$ for each degree of freedom (i spans from 1 to 57) are presented in (a) for η = 1.0 μPa·s, (b) for η = 3.0 μPa·s, and (c) for η = 18.4 μPa·s (air).

In Supplementary Fig. 6, we consider a square lattice with N = 9 spheres trapped and bounded by a standing wave composed of four counter-propagating plane waves, as shown in Supplementary Fig. 6a–c. The force matrix $\overleftrightarrow{\mathbf{K}}$ for this square lattice is still non-Hermitian, but the eigenvalues of the particular configuration chosen are all real, indicating that there is stable optical binding of the particles regardless of the viscosity (η). Interestingly, the $\frac{1}{2}m\left\langle v_i^2\right\rangle$ for certain degrees of freedom can be less than $k_{\mathrm{B}}T/2$, and the total kinetic energy of individual spheres can be less than $\frac{3}{2}k_{\mathrm{B}}T$, as demonstrated in Supplementary Fig. 6d–f. However, the average kinetic energy for the entire system $\frac{1}{3N}\sum_{i=1}^{3N}\frac{1}{2}m\left\langle v_i^2\right\rangle$ is always greater than $\frac{1}{2}k_{\mathrm{B}}T$, due to the non-Hermitian force field. Because of the degeneracies resulting from rotational and mirror symmetries, only seven lines are presented in Supplementary Fig. 6a–c.

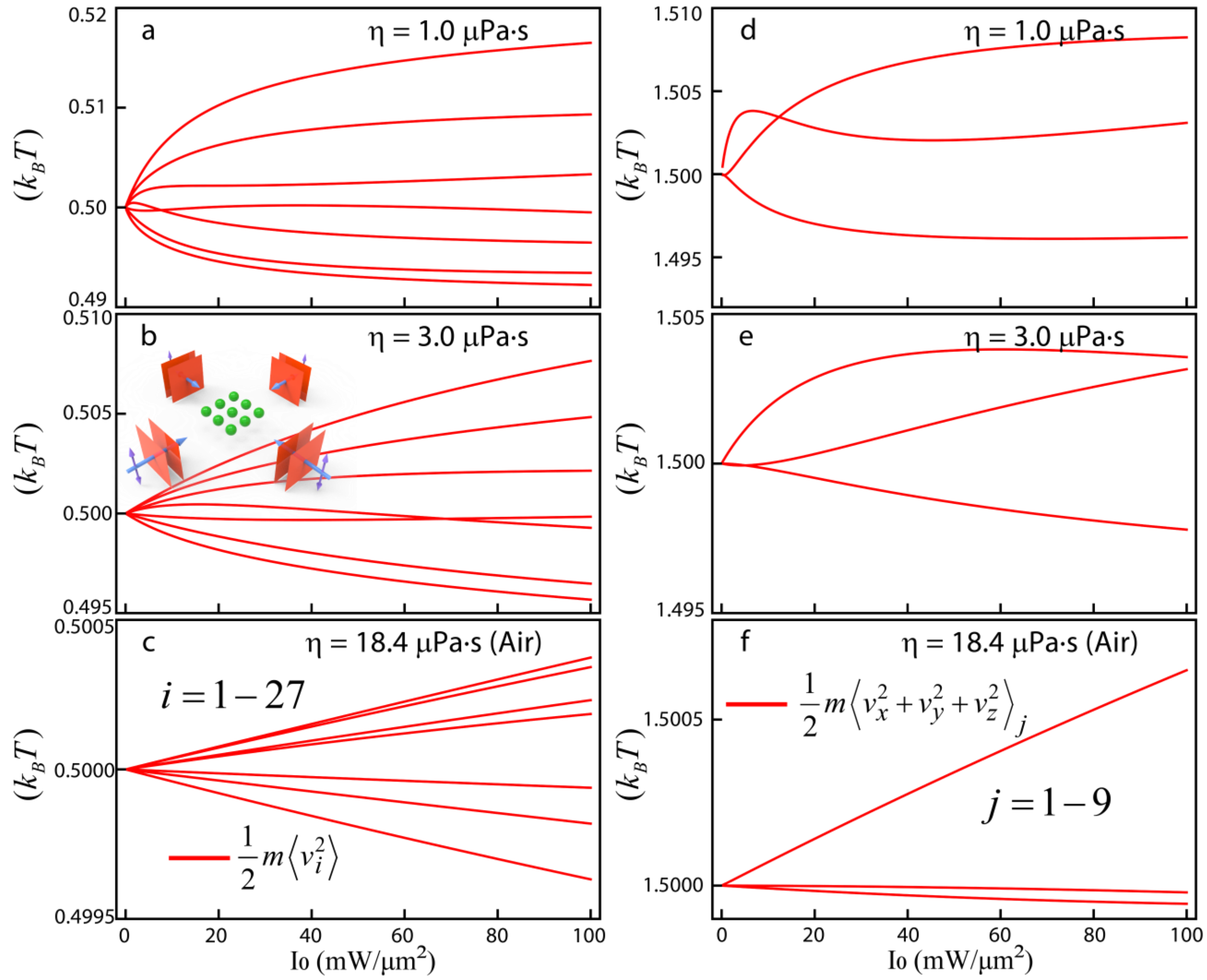

Supplementary Fig. 6| Approximate multi-particle NHNE theory for optical binding of N = 9 spheres (square lattice). The nine spheres each have a radius a = 0.2 μm and a refractive index n = 1.2, and are optically trapped and bounded at the high-intensity spots produced by the four linearly polarized plane waves (λ = 1.064 μm) in a low vacuum or in air, as illustrated in the inset in (b). Each plane wave has an intensity of I_0. As I_0 varies from 1 to 100 mW/μm^2, the kinetic energies $\frac{1}{2} m \langle v_i^2 \rangle$ for each degree of freedom (i spans from 1 to 27) are presented in (a) for η = 1.0 μPa·s, (b) for η = 3.0 μPa·s, and (c) for η = 18.4 μPa·s (air). The total kinetic energies $\frac{1}{2} m \langle v_x^2 + v_y^2 + v_z^2 \rangle_j$ for each single particle (j spans from 1 to 9) are presented in (d) η = 1.0 μPa·s, (e) for η = 3.0 μPa·s, and (f) for η = 18.4 μPa·s (air).

Supplementary Note 8: Size-dependency of an optically trapped Brownian particle

The non-Hermiticity of optical trapping depends strongly on the particle radius (a). We conducted new simulations involving a dielectric particle with a refractive index of n = 1.57 being optically trapped by two counter-propagating focused circularly polarized beams, as depicted in Supplementary Fig. 7a. To illustrate the impact of particle radius, we present the components and eigenvalues of the corresponding force matrix in Supplementary Fig. 7b and 7c, respectively, while varying the particle radius from a = 0.1 μm to 0.6 μm. Unlike the trapping stiffnesses k_{xx} and k_{yy}, which grow roughly in a monotonical manner with respect to the particle radius, the non-Hermitian force matrix component g, does not exhibit a monotonic increase with respect to the particle radius. Notably, g is small for a small radius, owing to the weak scattering force associated with Rayleigh particles. g is relatively large for a = 0.3 to 0.5 μm, and then drops again.

Supplementary Fig. 7d-e and 7f-g display the kinetic and potential energies, respectively, for different viscosities: η = 3.0 μPa·s and η = 18.4 μPa·s (Air). It is worth noting that in addition to optical force, the particle mass also has a significant impact (as depicted by Eq. (5) in the main text).

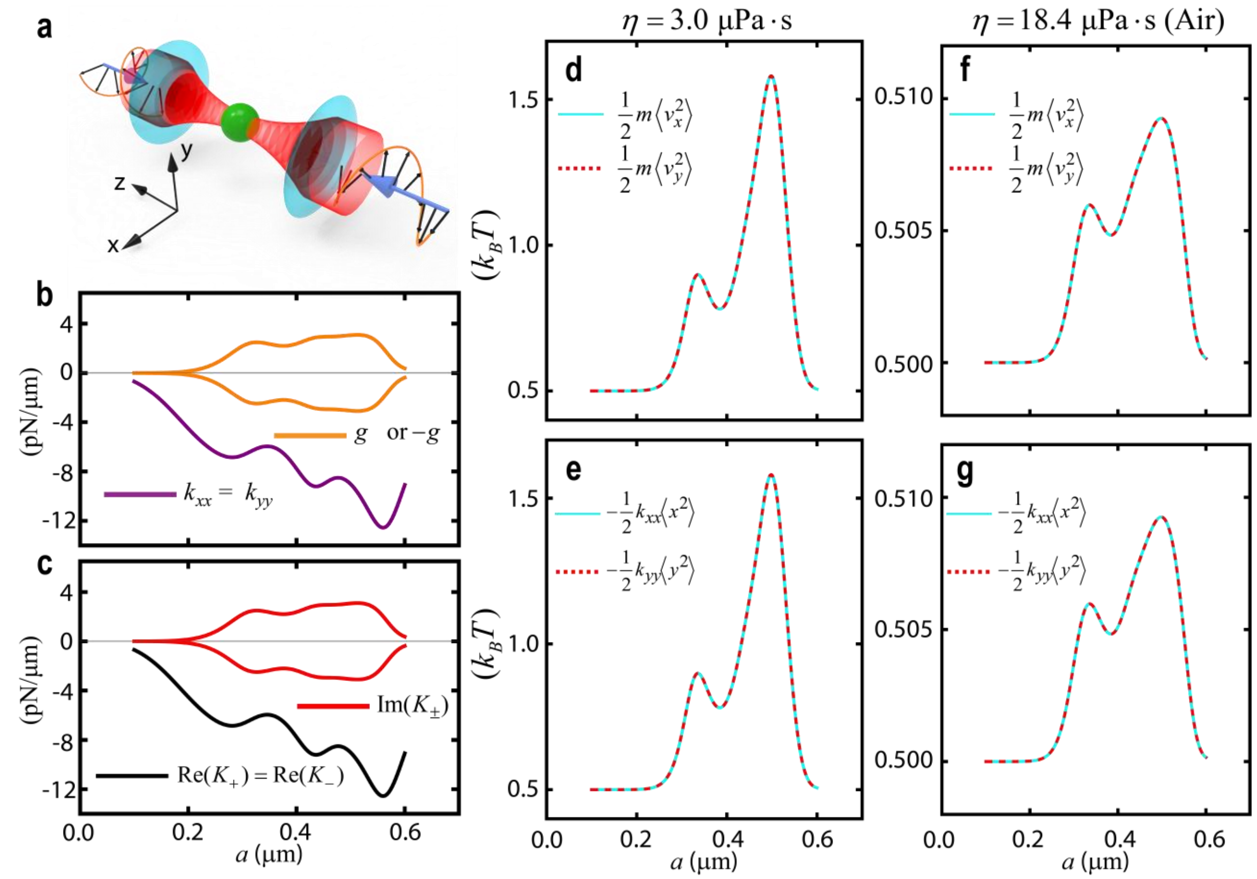

Supplementary Fig. 7| Influence of particle radius on the non-Hermitian non-equipartitioning in optical trapping. (a) A dielectric Brownian particle with a refractive index n = 1.57 is optically trapped by two counter-propagating focused beams (with wavelength λ = 1.064 μm and circular polarization). The focused beam has a numerical aperture of 0.9 and a filling factor of 1.0, and the beam power for each beam is normalized to 1.0 mW. (b) Components of the force matrix $\overleftrightarrow{\mathbf{K}}_{\mathrm{2D}} = \begin{bmatrix} k_{xx} & g \\ -g & k_{yy} \end{bmatrix}$ and (c) its eigenvalues versus radius a. For different viscosities, η = 3.0 μPa·s (d–e) and η = 18.4 μPa·s (f–g), average energies $\frac{1}{2}m\langle v_x^2\rangle$, $\frac{1}{2}m\langle v_y^2\rangle$, $-\frac{1}{2}k_{xx}\langle x^2\rangle$, and $-\frac{1}{2}k_{yy}\langle y^2\rangle$ are presented as lines.

Supplementary Note 9: Generating the saddle potential via coating a uniform dielectric layer on a gold sphere in optical trapping

As an additional instance of saddle potential, we examine the scenario where a uniform layer of dielectric is coated on a gold sphere for the purpose of optical trapping, as illustrated in Supplementary Fig. 8.

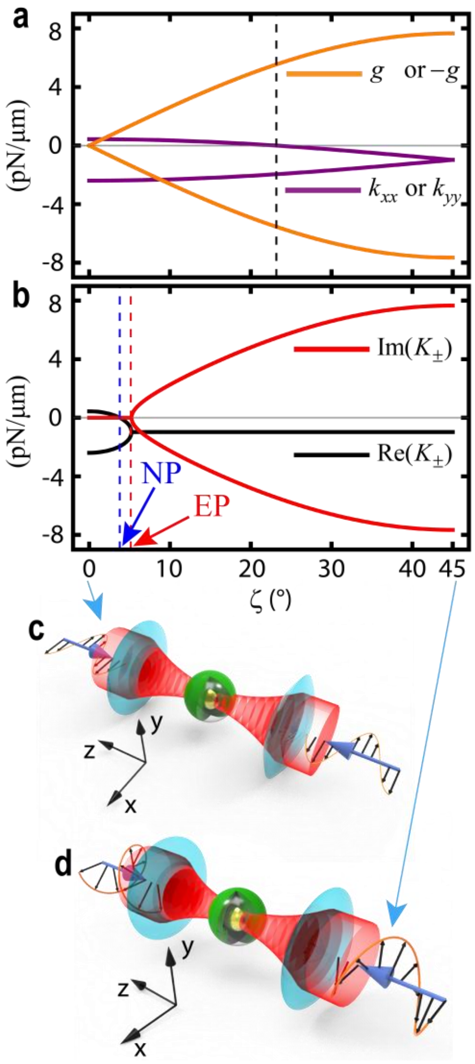

Supplementary Fig. 8| Generating the saddle potential via coating a uniform dielectric layer on a gold sphere in optical trapping. Components of force matrix $\overleftrightarrow{\mathbf{K}}_{2D} = \begin{bmatrix} k_{xx} & g \\ -g & k_{yy} \end{bmatrix}$ (a) and its eigenvalues (b) versus the polarization ($\hat{\mathbf{p}} = \hat{\mathbf{x}}\cos\zeta + i\hat{\mathbf{y}}\sin\zeta$) of the incident beams (with wavelength λ = 1.064 μm), where ζ varies from 0° to 45°. The schematic for the linear and circular polarizations are shown in (c) and (d), respectively. The particle has an inner radius 0.272 μm and an outer radius a = 0.34 μm. The refractive index of the dielectric is n = 1.57, while the relative permittivity of Au is $\varepsilon_{\mathrm{Au}} = -48.45 + 3.60$. The left-hand side of the black dashed line in (a) corresponds to the saddle potential, where $k_{xx} \cdot k_{yy} < 0$. The blue dashed line in (b) indicates the neutral point (NP), where one of the eigenvalues $K_\pm$ is 0. The red dashed line in (b) indicates the exceptional point (EP), respectively. The focused beam has a numerical aperture of 0.9 and a filling factor of 1.0, and the power of each beam is normalized to 1.0 mW.

Supplementary References

i Chandrasekhar, S. Stochastic problems in physics and astronomy. *Rev. Mod. Phys.* **15**(1), 1 (1943).

ii Ng, J., Lin, Z. F. & Chan, C. T. Theory of optical trapping by an optical vortex beam. *Phys. Rev. Lett.* **104**, 103601 (2010).

iii Greiner, W., Neise, L. & Stöcker, H. Thermodynamics and statistical mechanics. *Springer Science & Business Media* (2012).

iv Saberi, I. & Gittes, F. Nonconservative forcing and diffusion in refractive optical traps. *JOSA B* **28**(10), 2369-2373 (2011).

v Ng, J., Lin, Z. F., Chan, C. T. & Sheng, P. Photonic clusters formed by dielectric microspheres: Numerical simulations. *Phys. Rev. B* **72**, 085130 (2005).

vi Meiners, J. C. & Quake, S. R. Direct measurement of hydrodynamic cross correlations between two particles in an external potential. *Phys. Rev. Lett.* **82**(10), 2211 (1999).

vii Burns, M. M., Fournier, J.-M. & Golovchenko, J. A. Optical binding. *Phys. Rev. Lett.* **63**, 1233 (1989).
