# Supplementary material for: Non-Hermitian non-equipartition theory for trapped particles
Source: Nat Commun. 2024 Mar 4;15:1963. doi: 10.1038/s41467-024-46058-5 (PMC10912716; doi:10.1038/s41467-024-46058-5)
Supplement: Supplementary file 3 — Description of Additional Supplementary Files [file 41467_2024_46058_MOESM3_ESM.pdf]

Description of Additional Supplementary Files

Showcase the trajectories of optically trapped particles under trapping or saddle potentials in a low-vacuum environment, corresponding to each case depicted in Figure 4 of the main text, through movies. Here, η denotes the viscosity, ζ characterizes the polarization ($\hat{\mathbf{p}} = \hat{\mathbf{x}}\cos\zeta + i\hat{\mathbf{y}}\sin\zeta$) of the beams, EP denotes the exceptional point, and NP denotes the neutral point.

Supplementary Movie 1: Trapping potential; η = 1.0 μPa·s; ζ = 0°-45°.

Supplementary Movie 2: Trapping potential; η = 3.0 μPa·s; ζ = 0°-45°.

Supplementary Movie 3: Saddle potential; η = 1.0 μPa·s; ζ = 0°-45°.

Supplementary Movie 4: Saddle potential; η = 3.0 μPa·s; ζ = 0°-45°.
